# The importance of adjusting for potential confounders in Bayesian hierarchical models synthesising evidence from randomised and non-randomised studies: an application comparing treatments for abdominal aortic aneurysms

**DOI:** 10.1186/1471-2288-10-64

**Published:** 2010-07-09

**Authors:** C Elizabeth McCarron, Eleanor M Pullenayegum, Lehana Thabane, Ron Goeree, Jean-Eric Tarride

**Affiliations:** 1 Department of Clinical Epidemiology and Biostatistics, McMaster University, Hamilton, Ontario, Canada; 2 Programs for Assessment of Technology in Health (PATH) Research Institute, St. Joseph's Healthcare Hamilton, Hamilton, Ontario, Canada; 3 Biostatistics Unit, St. Joseph's Healthcare Hamilton, Hamilton, Ontario, Canada

## Abstract

**Background:**

Informing health care decision making may necessitate the synthesis of evidence from different study designs (e.g., randomised controlled trials, non-randomised/observational studies). Methods for synthesising different types of studies have been proposed, but their routine use requires development of approaches to adjust for potential biases, especially among non-randomised studies. The objective of this study was to extend a published Bayesian hierarchical model to adjust for bias due to confounding in synthesising evidence from studies with different designs.

**Methods:**

In this new methodological approach, study estimates were adjusted for potential confounders using differences in patient characteristics (e.g., age) between study arms. The new model was applied to synthesise evidence from randomised and non-randomised studies from a published review comparing treatments for abdominal aortic aneurysms. We compared the results of the Bayesian hierarchical model adjusted for differences in study arms with: 1) unadjusted results, 2) results adjusted using aggregate study values and 3) two methods for downweighting the potentially biased non-randomised studies. Sensitivity of the results to alternative prior distributions and the inclusion of additional covariates were also assessed.

**Results:**

In the base case analysis, the estimated odds ratio was 0.32 (0.13,0.76) for the randomised studies alone and 0.57 (0.41,0.82) for the non-randomised studies alone. The unadjusted result for the two types combined was 0.49 (0.21,0.98). Adjusted for differences between study arms, the estimated odds ratio was 0.37 (0.17,0.77), representing a shift towards the estimate for the randomised studies alone. Adjustment for aggregate values resulted in an estimate of 0.60 (0.28,1.20). The two methods used for downweighting gave odd ratios of 0.43 (0.18,0.89) and 0.35 (0.16,0.76), respectively. Point estimates were robust but credible intervals were wider when using vaguer priors.

**Conclusions:**

Covariate adjustment using aggregate study values does not account for covariate imbalances between treatment arms and downweighting may not eliminate bias. Adjustment using differences in patient characteristics between arms provides a systematic way of adjusting for bias due to confounding. Within the context of a Bayesian hierarchical model, such an approach could facilitate the use of all available evidence to inform health policy decisions.

## Background

Health technology assessment has been defined as a multidisciplinary field of policy analysis studying the medical, social, ethical, and economic implications of development, diffusion, and use of health technology [1]. Evidence on the effects of interventions from comparative studies is a critical component of this process. The different types of study designs (e.g., randomised, non-randomised/observational) used to assess the effects of interventions can be arranged into a hierarchy, at the top of which is the randomised controlled trial (RCT) [2]. Randomisation increases the likelihood that the treatment groups will be balanced in terms of known and unknown prognostic or confounding variables. Consequently the treatment effects estimated from RCTs are less subject to the potential confounding effects of extraneous variables [3]. Evidence from RCTs alone, however, may not be sufficient to inform decision makers. In particular, the strict inclusion and exclusion criteria which are often applied in RCTs may limit their generalisability relative to non-randomised studies [4,5]. In some cases, compliance to randomisation, among the randomised studies, might also be an issue. Furthermore, the scarcity of randomised studies for certain non-drug technologies, such as medical devices and surgical procedures, may necessitate the use of evidence from non-randomised studies in addition to that available from randomised studies [4]. Contrary to ignoring evidence from non-randomised studies, it has been argued that all available evidence should be used to inform health care decision making [4-7]. Such an approach requires methods capable of synthesising evidence from both randomised and non-randomised studies.

Bayesian hierarchical modelling [5,8] has recently been proposed for synthesising evidence from randomised and non-randomised studies. Prevost et al. [5] applied their method to combine evidence relating to the relative risk for mortality from five randomised trials and five non-randomised studies evaluating mammographic screening. Other applications of Prevost's model include Grines et al. [9] and Sampath et al. [10].

As an extension to the model, Prevost et al. [5] proposed the inclusion of study covariates to explain differences in mean effects at the study type level. Although this is important, the authors did not model differences between study arms, which may be a limitation of this approach when dealing with non-randomised studies due to potential differences in baseline characteristics. Adjustment made using aggregate values will not account for potential imbalances between study arms resulting from the lack of randomisation. Another extension proposed by Prevost made use of a prior constraint, reflecting the assumption that evidence from non-randomised studies, having been derived from study designs with potential weaknesses [4], may be more biased than evidence from randomised studies. The effect of the prior constraint is to downweight the evidence from the non-randomised studies. This approach has been criticized as it may not eliminate bias [11].

The objective of this paper was to extend the Bayesian three-level hierarchical model developed by Prevost et al. [5] in order to accommodate the greater potential for bias among the non-randomised studies by adjusting study estimates for potential confounders using differences in patient characteristics between study arms. Modeling differences between study arms is important in order to correct for potential imbalances within studies which could bias the results.

We applied this new model to a subset of studies from a systematic review of endovascular (EVAR) and open surgical repair (OSR) in the treatment of abdominal aortic aneurysms (AAAs) [12]. The results were compared with those using covariates representing aggregate values for patient characteristics (e.g., mean age) within studies, as in Prevost et al. [5] and Sampath et al. [10], and with two approaches for downweighting biased evidence. Prevost's prior constraint to downweight the non-randomised studies was considered as well as an additional approach that combined a prior distribution based on the non-randomised studies with data from the randomised studies [8].

## Methods

### Prevost's original Bayesian three-level hierarchical model

The three-level Bayesian hierarchical model proposed by Prevost et al. [5] extends the standard two-level random-effects meta-analysis [13] to include an extra level to allow for variability in effect sizes between different types of evidence (e.g., randomised versus non-randomised study designs). In addition to variability between study estimates within each study type, this model has the capacity to deal with any added uncertainty due to study design [14]. The three levels allow for inferences to be made at the study, study type, and population levels. Although the model can accomodate more than two types of study designs, the application presented by Prevost et al. [5] combined evidence from two study types, randomised and non-randomised.

This model can be written as follows:(1)(2)(3)

(i = 1 or 2 for the 2 study types; j = 1,..., k_i _studies).

At the first level of the model (eq.1), y_ij _is the estimated log relative risk in the jth study of type i, which is normally distributed with mean ψ_ij _and variance s_ij_^2^. The ψ_ij _represent the underlying effect, on the log relative risk scale, in the jth study of type i. At the second level of the model (eq.2), the ψ_ij _are distributed about an overall effect for the ith type of study, θ_i  _with σ_i_^2 ^representing the between-study variability for studies of type i. At the third level of the model (eq.3) the study-type effects are distributed about an overall population effect, μ, with τ^2 ^representing the between-study-type variability.

To try to explain between study heterogeneity, Prevost et al. [5] extended their model to include a covariate for age at the study type level. This is shown in the equation below.(4)

In equation 4, x_ij _took the values of 0 and 1 for studies of women aged less than 50 years and studies of women 50 years and over, respectively. The same approach was used by Sampath et al. [10] to adjust for study covariates representing continuous variables such as average age and proportion of males in each study. Grines et al. [9] did not conduct covariate adjustment but rather used funnel plots to assess heterogeneity among individual study estimates.

### Extension of Prevost's model to adjust for imbalances between study arms

While heterogeneity refers to unexplained variation, bias refers to systematic deviations from the true underlying effect due, for example, to imbalances between study arms [2]. One potential source of bias is confounding [15], where an extraneous factor is associated with both the exposure under study (e.g., treatment) and the outcome of interest, but is not affected by the exposure or outcome [16]. Only when the groups being compared are balanced in all factors, both those that can be measured and those that cannot, that are associated with exposure and that affect the outcome (other than treatment) will it be certain that any observed differences between the groups are due to treatment and not the result of the confounding effects of extraneous variables. Randomisation increases the likelihood that the groups will be balanced not only in terms of the variables that we recognize and can measure but also in terms of variables that we may not recognize and may not be able to measure (i.e., unknowns) but that nevertheless may affect the outcome [3]. In contrast, the greater likelihood of imbalances within the non-randomised studies could have implications especially when combining both types of study designs. In order to deal with this problem, we extended Prevost's three-level model to adjust for differences within studies rather than adjusting for aggregate values at the study type level as in equation 4. The proposed approach uses the variation in imbalances across studies to adjust for differences in patient characteristics between treatment arms within studies. As with RCTs, the resulting balance in patient characteristics within studies should avoid the influence of confounding.

The following presents an extension of Prevost's model based on odd ratios, but could be extended to relative risk. This analysis was undertaken using a binomial model in which the odds of the event (e.g., death) are calculated for each study and study arm level information is incorporated in the model. The model can be written as follows:(5)(6)(7)(8)

(i = 1 or 2 for the 2 study types; j = 1,..., k_i _studies, m = 1,.., M confounders).

It is assumed that the number of events in each arm in the jth study of type i (i.e., r_Cij _and r_Tij _for control (C) and treatment (T), respectively) follows a binomial distribution defined by the proportion of patients who experience the event in each arm in the jth study of type i (i.e., p_Cij _and p_Tij_) and the total number of patients in each arm in the jth study of type i (i.e., n_Cij _and n_Tij_), as shown in equation 5. Equation 6 describes the log odds for the event in the control (γ_ij_) and treatment (γ_ij _+ ψ_ij_) arms of each of the k_i _studies.

This model assumes that the log odds ratio, ψ_ij_, follows a normal distribution with a mean which is the sum θ_i _(i.e., the overall intervention effect in the ith type of studies) and a study specific bias adjustment, α_m_(x_mTij _– x_mCij_), that is proportional to the relative differences between the study arms in each of the studies (eq.7). In this expression, x_mTij _and x_mCij _are the values of the m-th potential confounder in each of the study arms (i.e., treatment and control) in the jth study of type i while α_m _represents the mean bias for the m-th potential confounding variable, across all the studies. The remaining variables were defined as before.

Prior distributions for the unknown parameters were intended to be vague. Normal priors with mean zero and variance 0.26 truncated to be positive, were specified for both random-effects standard deviations (σ_i_,τ). The priors for σ_i _and τ corresponded to the priors used in Grines et al. [9] as they represented what may be considered reasonable priors in many situations [13]. These priors support equality between studies while discounting substantial heterogeneity. A Normal prior with mean zero and variance ten was used for the overall population effect (μ). Vague Normal priors with mean zero and variance 1000 were assigned to the log odds (γ_ij_'s). These priors were applied to generate results both adjusted and unadjusted for potential confounders. In addition to these priors, the adjusted model also required priors for the bias coefficients (α_m_) for each of the m-th potential confounders. These were also given vague Normal prior distributions with mean zero and variance 1000.

### Alternative methods for potentially biased evidence

For comparison purposes, we also considered two approaches proposed to downweight the evidence from non-randomised studies. This is generally done by increasing the variance. The first method considered was the prior constraint used by Prevost et al. [5] to assess the influence of the assumption that the randomised studies were less biased than the non-randomised studies, and hence that |μ - θ _1_| < |μ - θ _2_|. This approach increased the relative proportion of the between-study-type variance (τ ^2^) associated with the non-randomised studies compared to the randomised studies. In so doing the interpretation of μ is altered. Since the constraint gives more weight to the randomised studies, μ no longer represents the total population studied. The overall effects in the randomised and non-randomised studies are represented by θ_1 _and θ_2_, respectively. The second approach was the informative prior distribution used by Sutton et al. [8] which included the evidence from the non-randomised studies via the prior for the treatment effect and combined this with a likelihood based only on the data from the randomised studies. Sutton et al. [8] centred their informative prior for the population mean on the non-randomised pooled estimate but used a variance four times larger than that of the randomised studies. The same approach was used for the current analysis, hence an informative Normal(-0.5619,0.8179) prior distribution was specified for μ. The same prior distributions as previously specified were used for the other unknown parameters.

### Analyses

All of the analyses were conducted using MCMC simulation implemented in WinBUGS 1.4.3 software [17]. A 'burn-in' of 100 000 iterations was followed by a further 100 000 iterations during which the generated parameter values were monitored and summary statistics such as the median and 95% credible interval of the complete samples were obtained. History plots, autocorrelation plots, and various diagnostics available in the package Bayesian Output Analysis [18], performed on two chains, were used to assess convergence. See additional file 1: Appendix for WinBUGS codes. The data are available from the author upon request.

### Illustration

#### Data

Data from a previously published systematic literature review evaluating EVAR and OSR in the treatment of AAAs [12] were used to illustrate the impact of adjusting for imbalances between study arms when combining evidence from randomised and non-randomised studies. The review identified 79 comparative studies of which four were randomised and 75 were non-randomised. One of the primary outcomes was 30-day mortality reported as an odds ratio.

Evidence of the relative imbalances within the randomised and non-randomised studies, together with information on the predictors of perioperative mortality in patients undergoing OSR, from several risk scoring methods (e.g., Leiden score) [19], were used to inform the choice of covariates for adjustment in both the base case scenario and sensitivity analyses. No adjustment was made for imbalances in the original study [12]. The extent to which some covariate data were missing was also considered in an additional sensitivity analysis, in which values for the missing covariates were imputed.

### Base case scenario

In the base case analysis, the results were adjusted for imbalances in age, gender, and cardiac disease. For all three covariates imbalances were greater among the non-randomised studies. The three covariates were available in a total of 44 studies, four randomised and 40 non-randomised. A description of the data is given in Table 1.

**Table 1:** Covariate Data: Average Imbalance between Study Arms

	Base Case 3 Covariates^a^ (k = 4 randomised and 40 non-randomised)	Imputed data 5 Covariates^b^ (k = 4 randomised and 75 non-randomised)
Study Type Non-randomised	Average Difference (EVAR-OSR)	Average Difference (EVAR-OSR)
Male (proportion)	0.09	0.10
Age (years)	2.40	2.53
Cardiac disease (proportion)	0.12	0.14
Pulmonary disease (proportion)	not considered as missing in 43% of the 75 non-randomised studies	0.10
Renal disease (proportion)	not considered as missing in 54% of the 75 non-randomised studies	0.05
**Randomised**		
Male (proportion)	0.05	0.05
Age (years)	0.82	0.82
Cardiac disease (proportion)	0.05	0.05
Pulmonary disease (proportion)	not considered as missing in 25% of the 4 randomised studies	0.13
Renal disease (proportion)	not considered as missing in 50% of the 4 randomised studies	0.07

a.male, age, cardiac disease, b.male, age, cardiac disease, pulmonary disease, renal disease

### Sensitivity analyses

#### Priors

A sensitivity analysis was conducted to assess the impact of using different prior distributions for the between-study (σ_i_) and between-study-type (τ) standard deviations. The vague priors used in the base case analysis (σ _i_, τ ~ half-normal (0,0.51^2^)) were compared to the more informative yet "fairly unrestrictive" priors used by Prevost et al. [5] (σ _i _~ half-normal(0,0.36^2^), τ ~ half-normal(0,0.18^2^)) and to a set of less informative priors. The latter involved Normal truncated to be positive priors with mean zero and variance one for the between-study standard deviation for the randomised studies (σ_1_) and the between-study-type standard deviation (τ). A Uniform prior over the range (0,10) was specified for the between-study standard deviation for the non-randomised studies (σ_2_). The prior distributions for the other unknown parameters remained unchanged from the base case analysis.

#### Imputation for missing data

A second sensitivity analysis was conducted to use all the studies providing comparative information (i.e., 79 studies including four randomised) rather than a subset of studies (i.e., 44 studies including four randomised) and to adjust for additional covariates which could affect the 30-day mortality risk. Among the other risk factors used to predict mortality following AAA surgery, the Leiden and modified Leiden scores both included pulmonary and renal disease [19]. These may be particularly relevant in the current context, as imbalances in pulmonary and renal disease were found to be greater among the randomised studies than among the non-randomised studies [12].

Since all five covariates were present together in less than one third of all studies (i.e., two randomised and 23 non-randomised studies), missing covariate values were imputed. Multiple imputation was conducted using R 2.9.2 software [20] assuming that the covariates were missing completely at random.

This approach implemented the bootstrap method to first impute values for each missing variable by randomly selecting from the observed outcomes of that variable and then generated multiple imputations (three datasets) using iterative regression imputation, looping through until approximate convergence. The data are described in Table 1. The result was a single imputed dataset of 79 studies (four randomised and 75 non-randomised) which was then analysed, in WinBUGS, adjusting for imbalances in age, gender, cardiac disease, pulmonary disease, and renal disease. Results were generated using all three types of priors described in the sensitivity analysis.

## Results

### Base case scenario

#### Unadjusted for potential confounders

The four randomised and 40 non-randomised studies were first analysed separately without adjusting for differences in study arms using a standard Bayesian two-level hierarchical model [13] together with a Normal(0,10) prior distribution for the population mean and a Normal(0,0.26) truncated to be positive prior distribution for between-study standard deviation. This produced estimates of the pooled median odds ratio for the randomised studies alone of 0.32 (95% credible interval (CrI) 0.13,0.76) and for the non-randomised studies alone of 0.57 (95% CrI 0.41,0.82).

In comparison, the Bayesian three-level hierarchical model estimated the pooled median odds ratio for the randomised studies to be 0.43 (95% CrI 0.19,0.75) and for the non-randomised studies to be 0.54 (95% CrI 0.40,0.76). When randomised and non-randomised evidence was combined, the overall median odds ratio from the three-level model was 0.49 (95% CrI 0.21, 0.98). This comparison illustrates the effect of the three-level hierarchical model allowing the cross contribution of evidence between the randomised and non-randomised studies. As a result, the estimated odds ratios for the study types were drawn towards one another and the uncertainty associated with them was reduced. The relative discrepancy in the number of randomised and non-randomised studies resulted in the pooled estimate for the randomised studies being greatly influenced by the non-randomised studies' estimate. The odds ratio in the non-randomised studies however, was drawn in by a much smaller amount.

#### Adjusted for differences in age, gender and cardiac disease between study arms

Upon synthesising the randomised and non-randomised evidence, the three-level hierarchical model adjusting for imbalances between study arms in terms of age, gender and cardiac disease (eq.7) was applied to the data. Important differences were observed compared to the unadjusted analysis. Posterior median odds ratios were 0.35 (95% CrI 0.17,0.63) for the randomised studies and 0.39 (95% CrI 0.25,0.61) for the non-randomised studies. The overall estimated odds ratio was 0.37 (95% CrI 0.17,0.77).

'Naive' adjustments made using the mean age, proportion of males and proportion of patients with cardiac disease in each study, as in Prevost and Sampath [5,10], produced estimates of 0.57 (95% CrI 0.27,1.03) for the randomised studies and 0.62 (95% CrI 0.44,0.87) for the non-randomised studies. The estimated population odds ratio was 0.60 (95% CrI 0.28,1.20).

#### Alternative methods for potentially biased evidence

The prior constraint resulted in estimated posterior median odds ratios of 0.44 (95% CrI 0.20,0.76) and 0.54 (95% CrI 0.40,0.76), respectively for the randomised and non-randomised studies and an overall estimate of 0.43 (95% CrI 0.18,0.89). An informed prior distribution centred on the pooled estimate from the analysis of the non-randomised studies alone with a variance four times that of the randomised studies generated a single overall estimate of 0.35 (95% CrI 0.16,0.76).

**Figure 1:**
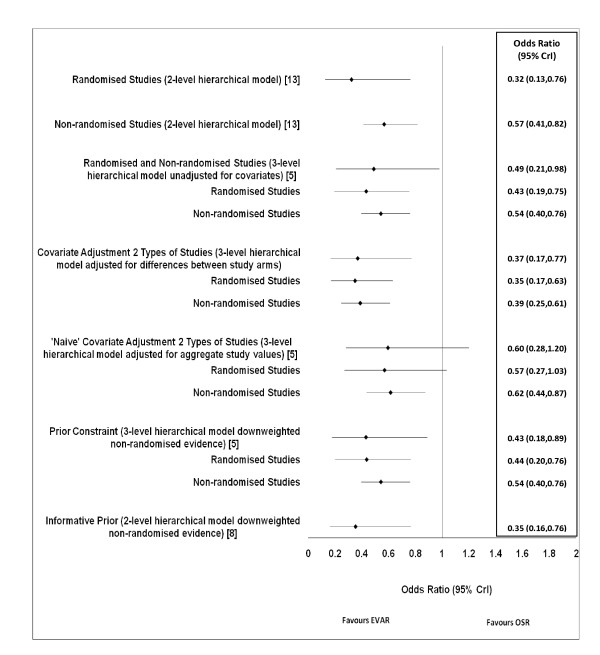
**Estimated overall (μ) and study type (θ _1_, θ _2_) odds ratios from Bayesian hierarchical models**. Perioperative mortality in studies of EVAR and OSR for the treatment of abdominal aortic aneurysms (four randomised controlled trials and 40 non-randomised studies)

Figure 1 compares the estimated odds ratios obtained from separate analyses of each type of study design using a two-level Bayesian hierarchical model with a three-level Bayesian hierarchical model synthesising evidence from both types of designs. In addition the estimates obtained when adjusting for differences in age, gender and cardiac disease between study arms or using aggregate study values are also presented. Estimates resulting from approaches downweighting the non-randomised evidence are displayed as well. All odds ratios are described in terms of the numerically approximated (via MCMC) median value of their posterior distribution and the associated 95% Bayesian CrI.

### Sensitivity analysis

#### Priors

As shown in Table 2, all three sets of priors produced similar values for the study type effects θ _1 _(randomised),θ _2 _(non-randomised) and for the overall odds ratio μ, though the precision of the credible intervals varied. Our vaguest priors produced an overall estimate which was not statistically significant.

**Table 2:** Adjustment for Differences in Patient Characteristics between Study Arms: Sensitivity to Prior Distributions

		Type of Prior		
Dataset	Posterior Estimate Median OR (95% credible interval)	Base Case Analysis: " Reasonably Vague" (Grines)	Sensitivity Analysis: "Fairly Unrestrictive" (Prevost)	Sensitivity Analysis: " Vaguest"
	**Overall (μ)**	0.37 (0.17,0.77)	0.37 (0.23,0.60)	0.37 (0.18,1.25)
**Base Case ** **3 Covariates^a ^** **(k = 44)**	**Randomised (θ_1_)**	0.35 (0.17,0.63)	0.36 (0.21,0.59)	0.34 (0.13,0.74)
	**Non-Randomised (θ_2_)**	0.39 (0.25,0.61)	0.38 (0.25,0.57)	0.40 (0.23,0.68)
	**Overall (μ)**	0.45 (0.20,0.95)	0.47 (0.28,0.74)	0.44 (0.13,1.31)
**Imputed** **5 Covariates^b ^** **(k = 79)**	**Randomised (θ_1_)**	0.42 (0.18,0.78)	0.46 (0.25,0.73)	0.39 (0.14,0.87)
	**Non-Randomised (θ_2_)**	0.49 (0.33,0.72)	0.49 (0.33,0.71)	0.49 (0.32,0.74)

a.male, age, cardiac disease, b.male, age, cardiac disease, pulmonary disease, renal disease

#### Imputed dataset

Adjustment for imbalances in pulmonary and renal disease in addition to age, gender and cardiac disease increased the estimated posterior median odds ratios for each of the study types and for the overall estimated odds ratio, though the inferences remained the same (Table 2).

## Discussion

We expanded the methods initially proposed by Prevost et al. [5] to take into account differences in patient characteristics between study arms. Comparison of the estimated odds ratios between the unadjusted three-level model, dominated by the 40 non-randomised studies, and the model adjusted using study arm differences revealed an overall odds ratio that had moved closer to the pooled estimate from the four randomised studies alone. The estimate was more precise than the randomised studies' estimate, reflecting the additional information from the adjusted non-randomised studies. 'Naïve' adjustments made using aggregate values in each study (centred about their respective mean values across all the studies) resulted in estimated odds ratios that were relatively closer to the pooled estimate from the non-randomised studies alone. The prior constraint proposed by Prevost et al. [5] did not alter the type level estimates to any noticeable extent. It did however change the contribution that each made towards the population level estimate. Relative to the unadjusted model, the introduction of the constraint resulted in a population level estimate which had moved towards the randomised studies' estimate both in terms of its location and precision. However, the shift and the precision of the credible interval were both less than when the model was adjusted for study arm differences. Sutton et al.'s [8] informative prior approach resulted in an overall odds ratio that was slightly closer to the randomised estimate than the model adjusted for imbalances. Its estimate was also slightly more precise.

All of the methods, with the exception of the model using aggregate study values for adjustment, produced population level estimates that had moved towards the randomised studies' estimate. While this lends credence to the ability of the extended model to adjust for potential confounders, this new model, in its current form, has some potential limitations. Because the imbalanced studies are adjusted, but not downweighted the credible intervals do not reflect the uncertainty due to this source of bias [15]. While downweighting itself may not eliminate bias, in conjunction with adjustment, it would give the biased studies less weight in the analysis. Ideally, this would be achieved by inflating the variances in such a way that, like the study specific bias adjustments, the downweighting was proportional to the relative differences between the study arms. Also, in its current form the proposed model does not address the extent to which variation in age, gender, and cardiac disease across studies may explain variation in study estimates. Rather, the objective of this study was to propose a method to adjust for differences in patient characteristics within studies, as a way of controlling for potential confounders. A practical limitation, as evidenced by this example, is the availability of arm level data from the primary papers. Any analysis could only be based on a subset of studies for which information on potential confounding variables happened to be available. This could bias the results if the observations were not missing at random [21]. Assuming that the covariates were missing completely at random the current analysis attempted to impute the missing values, though admittedly the two-stage nature of the current approach may appear inelegant (i.e., using R to impute the data and then analysing the new data in WinBUGS). A more natural solution would be to include the unobserved covariate values along with the unobserved parameters inside the MCMC, although this may add an additional layer of complexity. Due to the focus of the paper being Bayesian hierarchical models for combining randomised and non-randomised studies rather than methods to impute missing data, and for convenience, we decided to generate the missing values using R. Finally, adjustment cannot address the problem of unknown potential confounders [21].

Despite these limitations, we believe that the approach presented in this paper provides a systematic way of incorporating potentially biased evidence, relying on bias adjustment rather than arbitrarily downweighting the evidence. Prevost's and Sutton's approaches to downweighting assume the evidence from non-randomised studies is uniformly more biased, which, if there are well balanced non-randomised studies, may not necessarily be the case. Future research would be required to assess the generalisability of the proposed model beyond this single applied example. In particular, simulation studies would be necessary to ascertain its broader applicability. Part of the justification for combining evidence from both randomised and non-randomised studies rests on an all available evidence approach to health care decision making. The extent of missing covariate data in the current example suggests authors should be encouraged to better report the main characteristics of their study populations. The current example also illustrates the impact of different prior distributions on the precision of the results. The choice of prior could have implications in terms of informing health care decision making and may be particularly important in situations in which the data are not very informative [22].

## Conclusion

Synthesising evidence from both randomised and non-randomised studies requires methods for incorporating potential biases. In this paper, we propose a new approach to deal with bias due to confounding when combining randomised and non-randomised studies. This approach uses differences in patient characteristics to adjust for imbalances between study arms. Including aggregate study values for patient level covariates does not account for imbalances and downweighting may not eliminate bias. Within the context of a Bayesian hierarchical model the proposed approach could facilitate the use of all available evidence to inform health policy decisions.

## Competing interests

The authors declare that they have no competing interests.

## Authors' contributions

CEM conceived of the study, developed the model, analysed and interpreted the data, and drafted the manuscript. EMP conceived of the study, helped with statistical analysis, and critically reviewed the manuscript. LT conceived of the study, helped with statistical analysis, and critically reviewed the manuscript. RG conceived of the study, and critically reviewed the manuscript. JET conceived of the study, acquired the data, and helped with interpretation and drafting of the manuscript. All authors read and approved the final manuscript.

## Pre-publication history

The pre-publication history for this paper can be accessed here:

http://www.biomedcentral.com/1471-2288/10/64/prepub

## Supplementary Material

Additional file 1 **Appendix**. WinBUGS codes. Click here for file
