# Supplementary material for: The importance of adjusting for potential confounders in Bayesian hierarchical models synthesising evidence from randomised and non-randomised studies: an application comparing treatments for abdominal aortic aneurysms
Source: BMC Med Res Methodol. 2010 Jul 9;10:64. doi: 10.1186/1471-2288-10-64 (PMC2916004; doi:10.1186/1471-2288-10-64)
Supplement: Additional file 1 — Appendix. WinBUGS codes. [file 1471-2288-10-64-S1.PDF]

Appendix: WinBUGS Code

#Model
model{
for (j in 1:k){
#Likelihood for within-type model
rOSR[j] ~ dbin(pOSR[j],nOSR[j])
rEVAR[j] ~ dbin(pEVAR[j],nEVAR[j])
logit(pEVAR[j]) <- gamma[j] + psi[j]
logit(pOSR[j]) <- gamma[j]
gamma[j] ~ dnorm(0,0.001)
#Covariate Adjustment
psi[j] <- theta[type[j]] + alpha$_1$*(cadEVAR[j]-cadOSR[j]) + alpha$_2$*(maleEVAR[j]-maleOSR[j]) + alpha$_3$*
(ageEVAR[j]-ageOSR[j]) + (sigma[type[j]]*z[j]) **#Differences between study arms**
psi[j] <- theta[type[j]] + alpha$_1$*(cad[j]-cad.bar) + alpha$_2$*(male[j]-male.bar) + alpha$_3$* (age[j]-age.bar) +
(sigma[type[j]]*z[j]) **#Aggregate study values**
z[j] ~ dnorm(0,1)}
#Likelihood for between-type model
for (i in 1:2){
theta[i] <- mu + (tau*epsilon[i])
#Prior for base case
sigma[i] ~ dnorm(0,4)I(0,) **#Reasonably vague**
#Priors for sensitivity analysis
sigma[i] ~ dnorm(0,8)I(0,) **#Fairly unrestrictive**
sigma[1] ~ dnorm(0,1)I(0,) **#Vaguest**
sigma[2] ~ dunif(0,10)
epsilon[i] ~ dnorm(0,1)
OR.type[i] <- exp(theta[i])}
#Prior constraint
for (i in 1:1){
low.epsilon[1] <- min(epsilon[2],-epsilon[2])
up.epsilon[1] <- max(epsilon[2],-epsilon[2])
epsilon[1] ~ dnorm(0,1)I(low.epsilon[1],up.epsilon[1])}
for (i in 2:2){
low.epsilon[2] <- max(epsilon[1],-epsilon[1])
mod.epsilon2 ~ dnorm(0,1)I(low.epsilon[2],)
sign ~ dbern(0.5)
epsilon[2] <- (mod.epsilon2*sign) - (mod.epsilon2*(1-sign))}
alpha$_1$ ~ dnorm(0,0.001)
alpha$_2$ ~ dnorm(0,0.001)
alpha$_3$ ~ dnorm(0,0.001)
cad.bar <- mean(cad[])
age.bar <- mean(age[])
male.bar <- mean(male[])
mu ~ dnorm(0,0.1)
#Informative prior
mu ~ dnorm(-0.5619,1.2226)
#Prior for base case
tau ~ dnorm(0,4)I(0,) **#Reasonably vague**
#Priors for sensitivity analysis
tau ~ dnorm(0,30)I(0,) **#Fairly unrestrictive**
tau ~ dnorm(0,1)I(0,) **#Vaguest**
OR.overall <- exp(mu)}
